# 1,10-Secoguaianolides from *Artemisia austro-yunnanensis* and Their Anti-Inflammatory Effects

**DOI:** 10.3390/molecules23071639

**Published:** 2018-07-05

**Authors:** Lan Liu, Weifeng Dai, Cheng Xiang, Jun Chi, Mi Zhang

**Affiliations:** Faculty of Life Science and Technology, Kunming University of Science and Technology, Kunming 650500, China; 18213585697@163.com (L.L.); dwflove@163.com (W.D.); xcheng0871@163.com (C.X.); chijun16@126.com (J.C.)

**Keywords:** 1,10-secoguaianolide, anti-inflammation, *Artemisia austro-yunnanensis*, NF-*κ*B regulation, iNOS inhibition

## Abstract

Seven 1,10-secoguaianolides **1**–**7**, including a new one (compound **1**), were isolated from *Artemisia austro-yunnanensis* and identified by HRESIMS and other spectroscopic methods. Their anti-inflammatory effects were evaluated by the model of LPS-induced RAW264.7 cells in *vitro*. Bioassay results showed that six of them (**1**–**4**, **6** and **7**), with the exception of **5**, produce some cytotoxicity on RAW264.7 cells at its high dosage, can significantly decrease the release of NO, TNF-*α*, IL-1*β*, IL-6 and PGE2 in a dose dependent manner, and down-regulate the expression of proteins iNOS and COX-2. The mechanism study indicated they regulated the NF-*κ*B dependent transcriptional activity through decreasing the phosphorylation of NF-*κ*B. Further, the relationship between their structures and cytokines to anti-inflammatory were studied by PCA and discussed.

## 1. Introduction

Guaianolides are a kind of guaiane-type sesquiterpenes with lactone moieties, abundant in the plants from the families Compositae, Labiatae, Zingiberaceae, and so on. Based on the differences in the position of lactone moieties and carbon skeletons, guaianolides are classified into many types. Most of them belong to the 12,8-olide or 12,6-olide-guaiane types, and a few of them belong to the 12,5-olide-, 1,2-seco, 4,5-seco, 7,8-seco, or 1,10-seco-guaiane types [1,2,3,4,5,6]. Previous investigations showed that guaianolides possess broad pharmacological activities, including anti-cancer, anti-inflammation, antiparasitic, antiulcerogenic, antioxidant, antibacterial and antifungal effects [7,8,9,10,11], which make guaianolides target compounds in chemical studies of the abovementioned plants.

**Figure 1:**
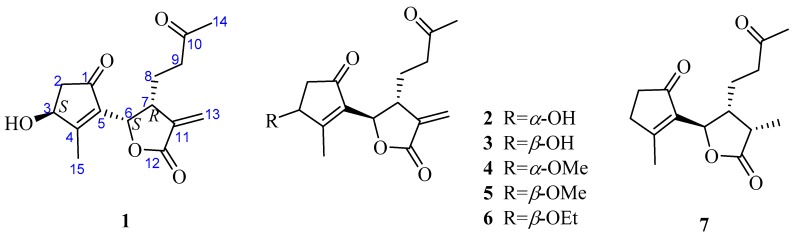
Chemical structures of compounds **1**–**7**.

*Artemisia austro-yunnanensis* Ling et Y. R. Ling is a semi-herbaceous shrub, belonging to the genus *Artemisia*, family Composite. It is usually distributed in the tropical and subtropical regions, such as the south of China, Thailand, Burma and Laos [12]. Previous phytochemical research by our group resulted in the isolation of more than fifty compounds from its whole plant [12,13,14], including 12,6-olide-guaiane sesquiterpenes, eudesmane sesquiterpenes, monocyclefarnesanes, nor-sesquiterpenes, coumarins, lignins, phenols, fatty acids, and steroids. However, guaianolides are still the main components in *A. austro-yunnanensis*. In order to further develop the guaianolides from this plant, a continuing studied was carried out and led us to obtain another seven 1,10-secoguaianolides **1**–**7**, including the new one **1** (Figure 1).

The traditional effects and structural similarities of natural compounds are the important evidence and guidance to bioactive research on natural constituents. Analysis of our isolates **1**–**7** displayed their structures are very similar to the iso-seco-tanapartholides, which are also obtained from *Artemisia austro-yunnanensis* in current study and it is reported that they act in the NF-*κ*B signaling pathway [15]. It is widely known that many molecules involved in the early and inflammatory responses of the immune response are regulated by the NF-*κ*B [16,17]. Therefore, the anti-inflammatory activity of those seven 1,10-secoguaianolides **1**–**7** was studied in this paper and the relationship between their structures and cytokines to anti-inflammatory activity were studied by PCA and discussed.

## 2. Results and Discussion

### 2.1. Structural Identification of Compounds

Compound **1** was obtained as a colorless oil. Its molecular formula was assigned as C_15_H_18_O_5_ with even indices of hydrogen deficiency by HRESIMS (*m*/*z* 279.1232 [M + H]^+^, calcd 279.1232). Its ^13^C-NMR and DEPT data displayed fifteen carbon signals, including two ketocarbonyl (*δ*_C_ 203.8, 208.3), a lactone carbonyl (*δ*_C_ 169.8), four olefinic carbons (*δ*_C_ 173.4, 138.2, 135.5, 123.2), two oxygenated secondary carbons (*δ*_C_ 72.4, 75.9), three methylene carbons (*δ*_C_ 23.8, 40.2, 43.0), one methine carbon (*δ*_C_ 42.0), and two methyl carbons (*δ*_C_ 14.9, 30.3). In the ^1^H-NMR spectrum of **1** (Table 1), two methyls at *δ*_H_ 2.12 (3H, s) and 2.25 (3H, s), and two oxymethines at *δ*_H_ 4.71 (1H, brs) and 5.45 (1H, d, 7.2 Hz), were observed. 

**Figure 2:**
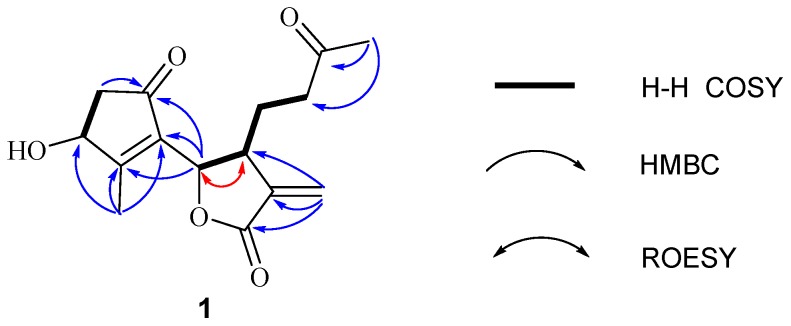
Key correlations of compound **1**.

**Figure 3:**
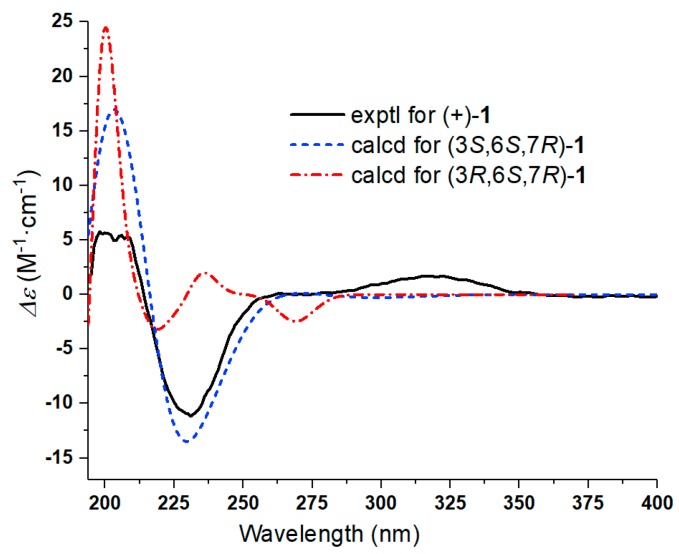
Comparison of the experimental ECD spectrum of (+)-**1** with calculated ECD spectra for (3*S*,6*S*,7*R*)-**1** (*σ* = 0.30 eV; shift = −16 nm) and (3*R*,6*S*,7*R*)-**1** (*σ* = 0.16 eV; shift = −38 nm) in MeOH. As a result, the absolute configurations of (+)-**1** was determined to be (3*S*,6*S*,7*R*)-**1**.

Further, two olefinic protons at *δ*_H_ 5.66 (1H, s) and 6.35 (1H, s), characteristic of an exocyclic olefin, were shown in the ^1^H-NMR, which were both correlated with an olefinic methylene carbon at *δ*_C_ 123.2 in the HSQC spectrum, indicating that **1** contained an exocyclic olefin that was positioned at C-11 by the HMBC correlations from those two protons to C-7, C-11 and C-12. Other HMBC correlations from H-2 to C-1, H-6 to C-1/C-4/C-5, Me-14 to C-9/C-10, and Me-15 to C-3/C-4/C-5, together with the ^1^H-^1^H COSY correlations H-2 to H-3, H-6 to H-7, H-7 to H-8, and H-8 to H-9, suggested **1** was a 1,10-secoguaianolide (Figure 2). Comparison of the 1D NMR data of **1** with two known compounds, epi-iso-seco-tanapartholide and iso-seco-tanapartholide [10], revealed they have similar planar structures. However, some difference among them were still observed, including the downfield shifts of H-6 at *δ*_H_ 5.45 and H-7 at *δ*_H_ 3.29 and their increased coupling constants of 7.2 Hz in **1**, which is assumed to result from the same orientations of H-6 and H-7. In the ROESY experiment, H-6 and H-7 were confirmed to be cofacial by their correlation peak. Further, the absolute configuration of **1** was determined by comparing the experimental electronic circular dichroism (ECD) spectrum with the calculated one (Figure 3) [18,19,20,21]. The calculated ECD curve of (3*S*, 6*S*, 7*R*)-**1** fully matched the experimental spectrum. Therefore, the structure of **1** was identified to be as shown in Figure 1.

The structures of compounds **2**–**7** were elucidated as epi-iso-seco-tanapartholide (**2**) [15], iso-seco-tanapartholide (**3**) [15], 3*α*-methoxy-3*β*-deshydroxy-iso-seco-tanapartholide (**4**) [22], iso-seco-tanapartholide-3-*O*-methyl ether (**5**) [22], 3*α*-ethoxytanapartholide (**6**) [23], and 3-deshydroxy-iso-seco-tanapartholide (**7**) [22,24] by comparing their HRESIMS and NMR data with the data reported in the corresponding references.

### 2.2. Cytotoxicity Assay by MTT Method

**Figure 4:**
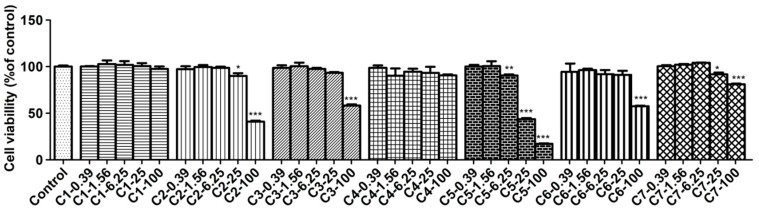
The effects of compounds on cell viability in LPS-induced RAW264.7 cells. RAW264.7 cells were treated with LPS (1 μg/mL) without or with compounds (0.39–100 μM) for 24 h. Cell viability was determined by MTT assay. Data are expressed as the mean value ± SD (*n* = 3). *** *p* < 0.001, ** *p* < 0.01, * *p* < 0.05 as compared to the control.

The cytotoxicity of compounds **1**–**7** on RAW 264.7 cells was examined by the MTT assay and the results are shown in Figure 4. It demonstrated that compounds **1** and **4** did not affect normal cell growth at concentrations up to 100 μmol/L, while **2**, **3**, **6** and **7** did not up to 25 μmol/L. However, compound **5** produced cytotoxicity to RAW264.7 cells at its concentration of 6.25 μmol/L so it was not subjected to further study (Figure 4). From above, the safe concentrations of compounds **1**–**4**, **6** and **7** used in anti-inflammatory study have to be lower than 25 μM.

### 2.3. Effects of Compounds on Inhibition against Nitric Oxide (NO) Production

The inhibitory effects of compounds against NO production were determined by measuring the level of NO accumulation in culture media, and l-NMMA and dexamethasone (DXM) were used as positive controls. As results, LPS (1 μg/mL) induced significant NO production compared with the naive control. All tested compounds (**1**–**4**, **6** and **7**) can inhibit NO production with IC_50_ values ranging from 0.79 to 31.53 μM (Table 2), especially compounds **2**–**4** (IC_50_ < 10 μM).

NO is an important physiological transmitter and chemical messenger in the body, whose overproduction will induce inflammatory responses and organism damage [25]. At present, the model of inhibiting NO production in LPS-stimulated RAW264.7 cells has been applied widely to screen the lead compound with anti-inflammatory activity [26]. Accordingly, inhibiting NO production has also been an important indicator to evaluate if the compound possesses potential anti-inflammatory activity and is worth further study. Based on our results in this part, those tested six compounds **1**–**4**, **6** and **7** exhibited favorable activities on inhibition of NO production, which is helpful to our future research work.

### 2.4. Effects of Compounds on Levels of TNF-α, IL-1β, IL-6 and PGE2 in RAW264.7 Cells

**Figure 5:**
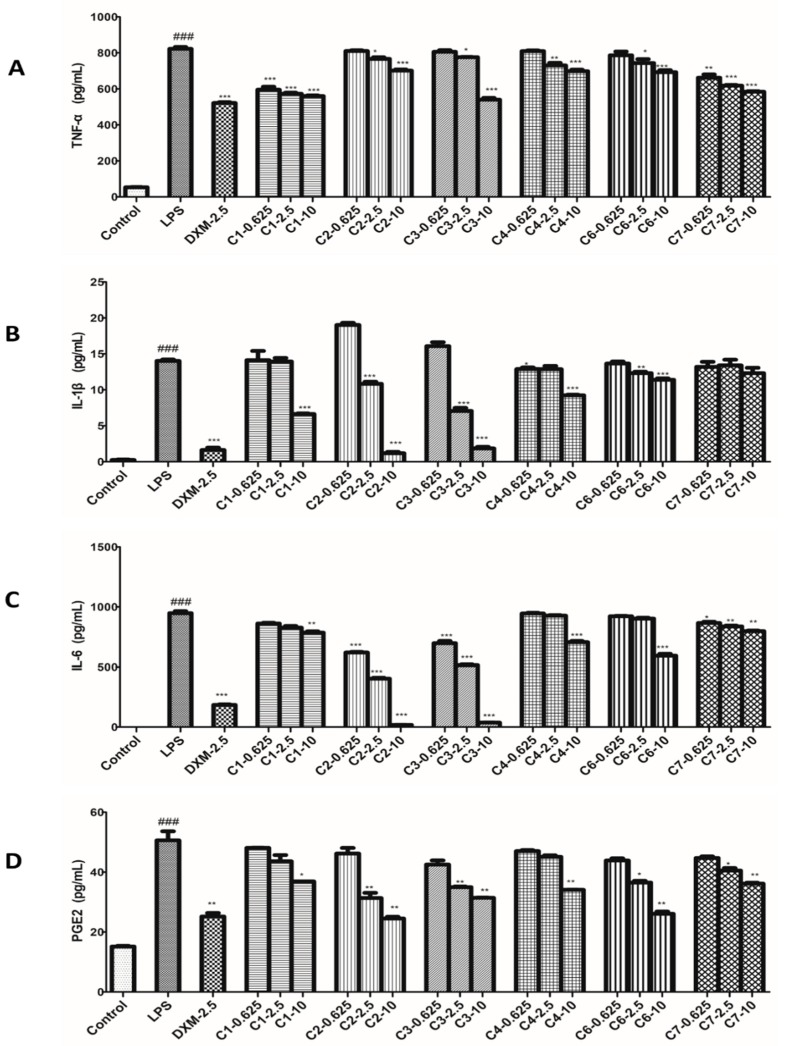
Effects of compounds on the TNF-*α*, IL-1*β*, IL-6 and PGE2 productions in RAW 264.7 macrophages. Cells were pretreated with compounds for 1 h and then stimulated with LPS (1 μg/mL) for 12 h. The productions of TNF-*α* (**A**), IL-1*β* (**B**), IL-6 (**C**) and PGE2 (**D**) were measured as described in the materials and methods. Data shown represent the mean value ± SD (*n* = 3). ^###^ *p* < 0.001 when compared with control versus LPS; *** *p* < 0.001, ** *p* < 0.01, * *p* < 0.05 as compared to the group treated with LPS alone.

ELISA assay was used to test the levels of TNF-*α*, IL-1*β*, IL-6 and PGE2 influenced by compounds on LPS-stimulated RAW264.7 cells. In this bioassay, RAW264.7 cells were treated with three concentrations of compounds and stimulated with LPS for 12 h, and the 2.5 μmol/L DXM was used as a positive control. The results showed those four inflammatory factors in LPS-induced RAW264.7 cells strongly increased compared with the untreated control cells. As expected, all tested compounds (**1**–**4**, **6** and **7**) and DXM reduced the production of TNF-*α*, IL-1*β*, IL-6 and PGE2 in a dose-dependent manner. As shown in Figure 5, the inhibition of each compound against those four factors was better at high dosage but was not good enough at low dosage. Correspondingly, at the medium dosage group, each of them produced moderate effect compared with other two groups.

Inflammatory response includes a series of complex biological reactions and is regulated by many inflammatory factors [27]. The most common inflammatory factors are NO, TNF-*α*, IL-1*β*, IL-6, and PGE2, etc., which participate in the inflammatory process of occurrence and development [28,29,30,31,32]. From the view of our bioassay in those factors, most of tested compounds produced significantly inhibitory effects at their medium or high dosages, and the effects in high dosage groups were best. However, at the same dosage, the tested compounds revealed different potencies on the same factor, indicating the ways and targets they acted cells were different.

### 2.5. Effects of Compounds on LPS-Induced iNOS and COX-2 Proteins in RAW264.7 Cells

**Figure 6:**
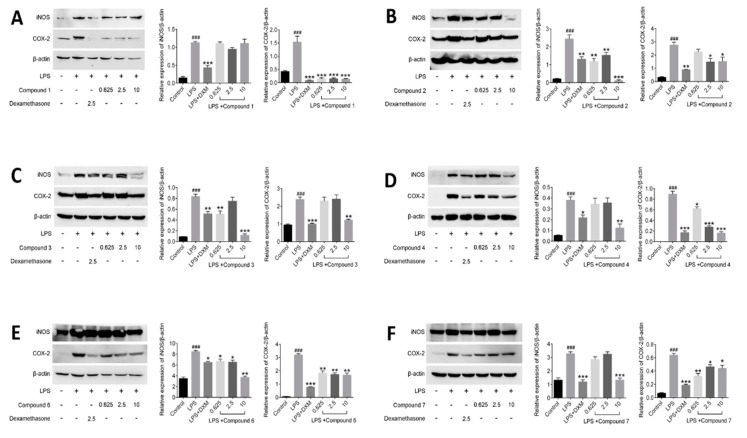
Effects of compounds on iNOS and COX-2 protein expressions in LPS-stimulated RAW264.7 cells. RAW264.7 macrophages were pretreated with various concentrations of compounds for 1 h prior to LPS (1 μg/mL) treatment. Using *β*-actin expression as an internal control. Data shown represent the mean value ± SD (*n* = 3). ^###^ *p* < 0.001 when compared with control versus LPS; *** *p* < 0.001, ** *p* < 0.01, * *p* < 0.05 as compared to the group treated with LPS alone.

In this assay, the levels of iNOS and COX-2 proteins in LPS-induced RAW264.7 cells exposed to three concentrations of compounds were examined, and 2.5 μmol/L DXM was used as a positive control. As shown in Figure 6, the expression of the iNOS and COX-2 proteins were barely detected in the non-stimulated cells. However, the levels of those two proteins increased markedly after the LPS treatment for 12 h. The result showed all tested compounds (**1**–**4**, **6** and **7**) displayed inhibition against the expression of iNOS and COX-2 proteases on the LPS-stimulated RAW264.7 cells.

Besides many inflammatory factors, the inflammatory response is related to many kinds of proteases [33]. Among these proteases, most attention has been paid to iNOS and COX-2 [34]. iNOS are mainly distributed in macrophages, liver cells, and chondrocytes, which can catalyze the synthesis reaction of NO. Generally, inflammatory response will induce overexpression of iNOS, so as to produce large amount of NO [35]. On the other hand, COX-2 is one of the two isoforms of COX, the other is COX-1 [36]. Their structures are very similar, but their expression pattern and function in organisms are quite different. Although both of them can catalyze the synthesis reaction of prostaglandins (PGs), previous investigation still suggested that prostaglandins (PGs) catalyzed by COX-1 have protective functions of the body and participate in normal physiological processes, while PGs catalyzed by COX-2 have correlated with inflammatory response [37]. The main reason is that COX-2 is usually to be expressed by stimulation, such as many kinds of chemical and physical damage, biological factors, etc. [38]. Based on the biological tests of the effects on NO and PEG2, the activities of compounds on the expression of iNOS and COX-2 in the LPS-stimulated RAW264.7 cells were carried on in this part. As results, the compounds **2**–**4** showed the better inhibitory effects against the expression of iNOS than that of other tested compounds at the dosage of 10 μM, consisting with the results in bioassay of NO, in which compounds **2**–**4** revealed better inhibition of NO production than others. However, the same tendency of effect has not been observed between COX-2 and PEG2. In theory, the PGE2 content changed resembled to those of COX-2. Actually, based on the results, the inhibition of compounds **2**–**4**, **6** and **7** against PEG2 are better than that of **1**, but the inhibition of compound **1** against expression of COX-2 are best among all tested compounds. It indicated that the action mechanisms of those compounds are quite different and deserved further study.

### 2.6. Effects of Compounds on Regulating NF-κB Activation in LPS-induced RAW264.7 Cells

**Figure 7:**
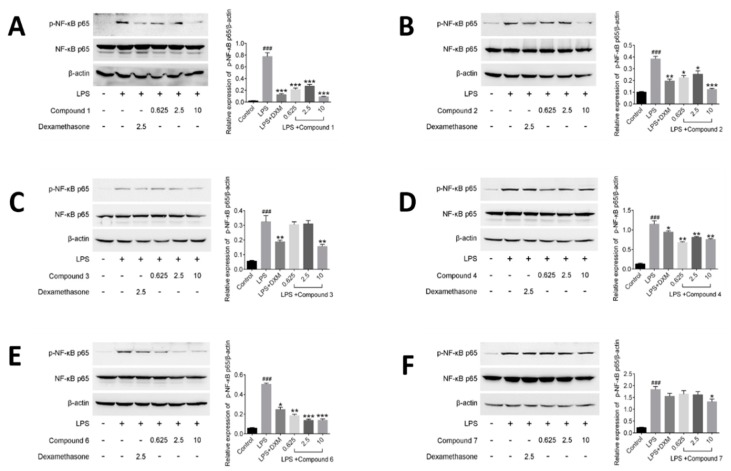
Effects of compounds on NF-*κ*B protein expression in LPS-stimulated RAW264.7 cells. RAW264.7 macrophages were pretreated with various concentrations of compounds for 1 h prior to LPS (1 μg/mL) treatment. Using *β*-actin expression as an internal control. Data shown represent the mean value ± SD (*n* = 3). ^###^ *p* < 0.001 when compared with control versus LPS; *** *p* < 0.001, ** *p* < 0.01, * *p* < 0.05 as compared to the group treated with LPS alone.

Compounds suppressing the phosphorylation of NF-*κ*B (active subunit p65) into the nucleus were investigated in this part. It was observed that p65 was mostly distributed in the cytoplasm and hardly translocated into the nucleus in unstimulated cells. But treatment with LPS for 30 min increased p65 protein level in the nucleus in model group. Meanwhile, it was observed that DXM and tested compounds **1**–**4**, **6** and **7** have certain impact on suppressing the nuclear translocation of p65, compared with LPS-induced cells in model group (Figure 7).

NF-*κ*B regulates precisely the expression of different genes through its different dimers, which play a critical role in the inflammatory processes and regulate the expression of inflammatory cytokines [30,39]. Among the dimers of NF-*κ*B, the most common is the p50/p65 heterodimer. In the resting cells, NF-*κ*B forms a complex with an inhibitor of NF-*κ*B (I*κ*B) in the cytoplasm. But when cells are induced by some external stimulus, such as LPS, cytokines and mitogens, I*κ*B will be phosphorylated and free NF-*κ*B to translocate into the nucleus. And then, the free NF-*κ*B interacts with *κ*B elements in the promoter regions of multiple pro-inflammatory genes, and induces the relevant gene expression [40]. In our assay, the compounds **1**–**4**, **6** showed the better inhibitory effects against the phosphorylation of NF-*κ*B than that of compound **7** at the dosage of 10 μM. In the previous test results, compound **7** was found to have inhibitory effect on inflammatory factors, but the effect was not as good as that of the other compounds, and dose-dependence was not obvious. As expected, the result of compound **7** on regulating NF-*κ*B activation in LPS-induced RAW264.7 cells was consistent with the previous test result and further illustrated that the anti-inflammatory effects of compounds **1–4** and **6** are superior to that of compound **7**. Nevertheless, the above results preliminarily revealed the potential mechanism of anti-inflammatory of those compounds are related to NF-*κ*B pathway.

### 2.7. Correlations between Tested Inflammatory Factors and Calculated Molecular Descriptors of Compounds ***1**–**4**, **6** and **7***

**Figure 8:**
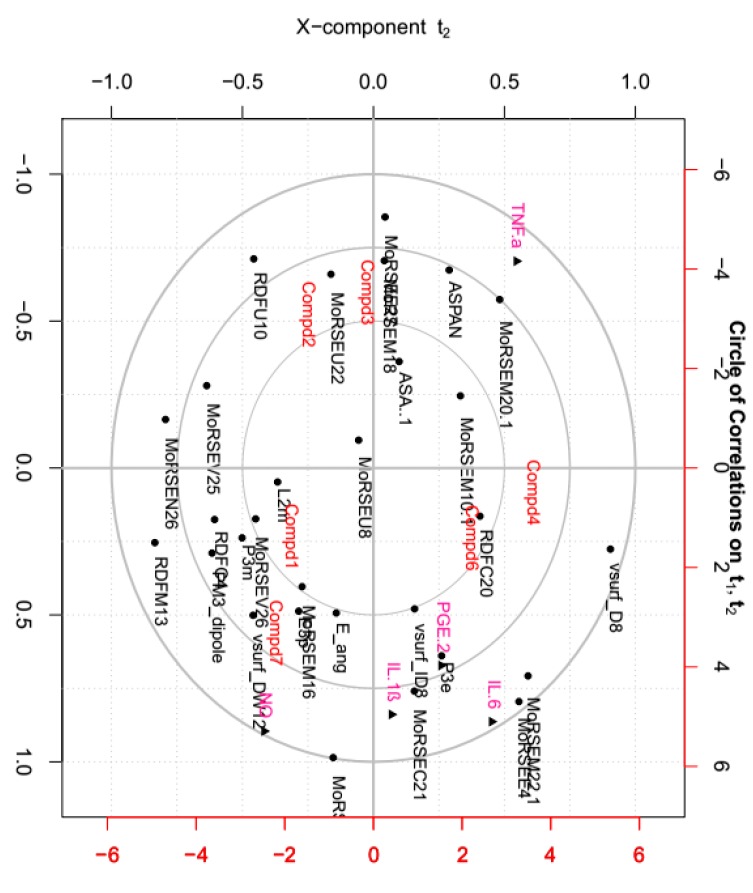
Principal component analysis (PCA) on relationship between inflammatory factors and calculated molecular descriptors presented by correlation circle.

Molecular descriptors encoding physic-chemical chemical properties and frameworks of chemical molecules are widely used in the investigation of chemoinformatics and quantitative structure activity relationships (QSAR) [41,42,43,44]. In this part, the relationships between above tested inflammatory factors and 29 molecular descriptors, including 18 3D-MoRSE descriptors, were analyzed by PCA and visualized in the biplot (Figure 8). As shown, many center correlations could be observed clearly: strongly positive correlations of IL-6/MoRSEE4 and MoRSEM22.1, IL-1*ß*/MoRSEC21, and PGE2/P3e. However, TNF-*α* was negative correlated with NO, IL-1*ß*, IL-6 and PGE2. Further, each tested compound displayed different potency on each inflammatory factor due to its structural difference, although they all possess same basic skeleton. It indicated that 3D-strcuture is very important in the bioactivity [45,46,47,48,49]. Taken together from PCA, the structural similarity between **2** and **3** is relatively higher than that of the others, and they displayed better effects against IL-1*ß*, IL-6 and PGE2 than the others. Meantime, the effects of compounds **1** and **7** were better against TNF-*α* but worse against production of NO than the others.

The structure-activity relationship study not only helps in the discovery of the lead compounds, but also benefits structural modification and optimization of those lead compounds. Generally, the presence of active functional groups is the key factor in active compounds. As early as 1960, it was found that the *α*-methylene *γ*-lactone moiety has the most important influence on the activities of guaianolides, resulting in better activities than those guaianolides without an *α*-methylene *γ*-lactone chemical moiety [50]. Additionally, with the help of the molecular descriptors used in QSAR, more detailed information and minor chemical structure differences can be obtained and correlated with their activities. Based on our study, the effect of each compound against those five inflammatory factors was different, suggesting different mechanisms of action on those factors that deserve further study.

## 3. Materials and Methods

### 3.1. General Experimental Procedures

Optical rotations were determined using a P-1020 spectropolarimeter (JASCO, Tokyo, Japan). UV spectra were measured on a UV-2401PC spectrophotometer (SHIMADZU, Kyoto, Japan). ECD spectra were measured on a Chirascan spectropolarimeter (APP, Leatherhead, UK). NMR spectra were recorded on an Ascend TM-800 MHz instruments (Bruker, Karlsruhe, Germany). HRESIMS spectra were obtained on a UPLC-Q-TOF (6530) spectrometer (Agilent, Santa Clara, CA, USA). Chromatographic separations was carried on the columns of silica gel (200–300 mesh, Qing Dao Ocean Chemical Factory, Qingdao, China), Sephadex LH-20 (20–100 μm, Pharmacia, Lambertville, NJ, USA), Sep-Pak C_18_ cartridges (Waters, Milford, MA, USA), ODS-C_18_ (40–63 μm, Fuji, Osaka, Japan), Agilent 1200 series HPLC, and semi-preparative HPLC (Beijing Chuangxintongheng Science & Technology Co., Ltd., Beijing, China), respectively.

### 3.2. Plant Material

The whole plant of *A. austro-yunnanensis* was collected in October 2012 from Sipsongpanna, Yunnan, China, and identified by Assistant Prof. Shishun Zhou of Xishuangbanna Tropical Botany Garden, Chinese Academy Sciences. The voucher specimen (AA-2012-10) was deposited in our laboratory of Faculty of Life Science and Technology, Kunming University of Science and Technology.

### 3.3. Extraction and Isolation

The dried whole plant (3.9 kg) of *A. austro-yunnanensis* was extracted with 95% EtOH (4 × 1.5 L × 5 h). The non-alcohol smelling residue was suspended in water and then extracted successively with petroleum ether (PE) and EtOAc to give 20.1 g of petroleum ether, 37.1 g of EtOAc, and 97.5 g of H_2_O soluble fractions, respectively. The EtOAc soluble fraction was subjected to silica gel column chromatography using gradients of PE-EtOAc (1:0, 10:1, 5:1, 1:1, 1:5, 0:1, *v*/*v*) to give eight fractions (Fr. a−h), monitored by TLC. Fr. c was subjected to ODS column chromatography (CC) with MeOH-H_2_O (20%→100%) and further purified by semi-preparative HPLC using MeOH-H_2_O (35%, 3 mL/min) to yield **6** (4.3 mg). Fr.d was chromatographed on Sephadex LH-20 with CHCl_3_-MeOH (1:1), to give Fr.d1−8. Fr.d2 was subjected to Sep-Pak C_18_ cartridges eluted with MeOH-H_2_O (20%→80%), and purified by semi-preparative HPLC with 25% MeOH-H_2_O, to yield **7** (1.1 mg). Fr.f was subjected to MCI gel, eluted successively with MeOH-H_2_O (20%→100%), to give Fr.f1−6. Fr.f6c was submitted to silica gel CC using hexane-EtOH (80:20) to yield **4** (7.4 mg) and **5** (3.5 mg). Fr. g was subjected to MCI gel, eluted successively with MeOH-H_2_O (20%→100%), to give Fr.g1−3. Fr.g3 was submitted to semi-preparative HPLC eluted with 15% MeOH-H_2_O and 10% CH_3_CN−H_2_O, successively, to yield **1** (1.3 mg), **2** (5.7 mg) and **3** (18.2 mg). Based on the isolates’ features determined by TLC and HPLC-DAD, 1,10-secoguaianolide analogues were not found in Fr. a, b, e, h of the EtOAc soluble fraction. The seven isolates **1**–**7** were stored at 4 °C for the characterization and bioactivity studies

### 3.4. Characterization of New Compound ***1***

*Compound***1**: colorless oil; [*α*]${}_{D}^{17}$ + 20.0 (*c* 0.13, MeOH); UV (MeOH) *λ*_max_ (log *ε*) 216 (3.20); CD (MeOH, *c* 0.55 × 10^−3^) *λ*_max_ (*ε*) 200 (+5.66), 231 (−11.16), 323 (+1.66); HRESIMS: *m*/*z* 279.1232 [M + H]^+^ (calcd for C_15_H_19_O_5_, 279.1232); ^1^H and ^13^C NMR data, see Table 1.

### 3.5. Samples and Reagents

LPS (*Escherichia coli* serotype 055: B5) and 3-(4,5-dimethylthiazol-2-yl)-2, 5-diphenyltetrazolium bromide (MTT), Dexamethasone, l-NMMA and Griess Reagent were purchased from Sigma-Aldrich (St Louis, MO, USA). Dulbecco’s modified Eagle’s medium (DMEM), fetal bovine serum (FBS) were obtained from Gibco BRL (Gaithersburg, MD, USA). TNF-*α*, IL-1*β*, IL-6 and PGE2 ELISA Kits were all purchased from Jiancheng Bioengineering Institute (Nanjing, China). Mouse *β*-actin, iNOS, COX-2 antibodies were purchased from Santa Cruz Biotechnology (Dallas, TX, USA). Rabbit p-P65 and p65 antibody were obtained from Cell Signaling Technology (Danvers, MA, USA). Anti-rabbit-HRP antibodies, anti-mouse-HRP antibodies and ECL detection kit were purchased from GE Healthcare (Waukesha, WI, USA). Other chemicals were of analytical grade and purchased from Sinopharm Chemical Reagent Co., Ltd. (Shanghai, China).

### 3.6. Cell Culture

RAW264.7, a mouse macrophage cell line, was purchased from the Cell Bank of the Shanghai Institute of Cell Biology and Biochemistry, Chinese Academy of Sciences (Shanghai, China), and cultured in high glucose DMEM supplemented with 10% heat-inactivated FBS, 100 U/mL penicillin and 100 μg/mL streptomycin in a 37 °C humidified incubator containing 5% CO_2_.

### 3.7. Cell Viability by the MTT Assay

MTT assay was used to evaluate the effect of those compounds on cell viability [51]. In brief, RAW264.7 cells were seeded in 96-well plates (Corning Inc., Corning, NY, USA) at a density of 2 × 10^4^ cells/well. After overnight growth, cells were treated with various concentrations of compounds (0.39−100 μM) for 1 h, followed in the presence or absence of LPS (1 μg/mL) for the next 24 h. 20 μL of MTT solution (5 mg/mL) was added and the cells were further cultured for 4 h. After that, the supernatant was carefully removed and then the resulting formazan crystals were dissolved in 150 μL DMSO with horizontal shaking. The absorbance at 570 nm (ref. 630 nm) was measured with a microplate reader (Molecular Devices, San Jose, CA, USA).

### 3.8. Assay for NO Production

To determine levels of NO in the cultured cells, RAW264.7 cells were seeded in 96-well plates (Corning Inc.) at a density of 8 × 10^4^ cells/well. After overnight growth, cells were treated with various concentrations of compounds (0.39−100 μM), l-NMMA or DXM [52] for 1 h followed in the presence or absence of LPS (1 μg/mL) for the next 12 h. Griess reagents were used to determine nitrite levels in the media. Briefly, an equal volume (70 μL) of supernatant was mixed with Griess reagent, and absorbance was measured at 540 nm against a standard sodium nitrite curve using a microplate reader (Molecular Devices).

### 3.9. Assay for TNF-α, IL-1β, IL-6 and PGE2 Levels

RAW264.7 cells were seeded into 24-well plates at a density of 5 × 10^5^ cells/mL and cultured overnight. After pretreatment with compounds (0.625 μM, 2.5 μM and 10 μM) for 1 h, the cells were stimulated with LPS (1 μg/mL) for 12 h and DXM was served as positive control [28]. The concentration of the release of TNF-*α*, IL-1*β*, IL-6 and PGE2 in the cell supernatants were assayed using ELISA kits (R&D Systems, Minneapolis, MN, USA) according to the manufacturer’s instructions. The concentrations were calculated from their standard curves, respectively.

### 3.10. Western Blot Analysis

Aliquots of the protein samples were separated on acrylamide gel by sodium dodecyl sulfate-polyacrylamide gel electrophoresis (SDS-PAGE) and electrophoretically transferred to nitrocellulose (NC) membranes (Millipore, Billerica, MA, USA). After blocking with 5% skimmed milk, the membranes were incubated with specific primary antibodies overnight at 4 °C. After rinsing, the membranes were incubated with a HRP-labelled secondary antibody containing a blocking solution for 1 h at room temperature. Bands were detected using enhanced chemiluminescence (ECL) reagents (GE Healthcare, Piscataway, NJ, USA) according to the manufacturer’s instructions. The immunosignals were captured using the Gel DOC™XR+system (BioRad Laboratories, Hercules, CA, USA) and densitometric data were studied following normalization to the housekeeping loading control.

### 3.11. Statistical Analysis

Results were expressed as mean ± SD (standard deviation). Statistical analysis was performed using one-way ANOVA followed by Tukey multiple comparison tests using GraphPad Prism (GraphPad Software Inc., San Diego, CA, USA). Level of significance was set at 0.05. Twenty nine molecular descriptors were selected and calculated using the semi-empirical AM1 method with MOE software to characterize the structures of tested compounds. The definition of each symbol was listed in Table S1 in the Supporting Information. The correlation between effects of tested compound against inflammatory factors and the calculated molecular descriptors were determined by principal component analysis (PCA) and performed on MATLAB software.

## 4. Conclusions

The present study indicated six 1,10-secoguaianolides **1**–**4**, **6** and **7** isolated from *Artemisia austro-yunnanensis* produced obvious anti-inflammatory effects via decreasing the release of NO, TNF-*α*, IL-1*β*, IL-6 and PGE2 and down-regulating the expression of proteins iNOS and COX-2. They regulated the NF-*κ*B dependent transcriptional activity through decreasing the phosphorylation of NF-*κ*B.

## Figures and Tables

**Table 1:** ^1^H- and ^13^C-NMR data of compound **1** (*δ* in ppm, *J* in Hz, CDCl_3_).

No.	^1^H NMR *^a^*	^13^C NMR *^a^*
1		203.8
2	2.34, m	44.2
	2.84, ddd (18.8, 6.7, 3.1)	
3	4.71, brs	72.4
4		173.4
5		135.5
6	5.45, d (7.2)	75.9
7	3.29, m	42.0
8	1.62, m	23.8
9	2.32, m	40.2
	2.48, m	
10		208.3
11		138.2
12		169.8
13	5.66, s	123.2
	6.36, s	
14	2.12, s	30.3
15	2.25, s	14.9

***^a^***^1^H-NMR was measured at 800 MHz, and ^13^C-NMR was measured at 200 MHz.

**Table 2:** Inhibitory effects of compounds against LPS-induced NO production in RAW264.7 Cells.

Compound	IC_50_ (μM)	SD
**1**	15.39	3.16
**2**	1.62	0.20
**3**	0.79	0.13
**4**	7.63	1.99
**6**	19.32	0.22
**7**	31.53	5.07
l-NMMA	24.95	1.46
DXM	0.20	0.98

Data shown represent the mean value ± SD (*n* = 3).

## Supplementary Materials

1D NMR, 2D NMR and HRESIMS of **1**, and the symbols and definitions of the calculated molecular descriptors of compounds **1**–**7** (Table S1) are available free of charge on the Internet.

## Abbreviations

HRESIMS	high resolution electrospray mass spectroscopy
NMR	nuclear magnetic resonance spectrometer
^1^H-^1^H COSY	^1^H-^1^H chemical-shift correlation spectroscopy
HSQC	heteronuclear single quantum coherence
HMBC	heteronuclear multiple-bond correlation
ROESY	rotating-frame overhauser effect spectroscopy
ECD	electronic circular dichroism
LPS	lipopolysaccharide
l-NMMA	l-N^G^-monomethyl arginine citrate
DXM	dexamethasone
NO	nitric oxide
IL-1*β*	interleukin-1*β*
IL-6	interleukin-6
TNF-*α*	tumour necrosis factor-*α*
iNOS	inducible NO synthase
COX-2	cyclooxygenase-2
NF-*κ*B	nuclear factor-*κ*B
PGE2	prostaglandin E2
ELISA	enzyme-linked immunosorbent assay
WB	western blot
SDS-PAGE	sodium dodecyl sulfate-polyacylamide gel electrophoresis
MTT	3-(4,5-dimethyl-2-thiazolyl)-2,5-diphenyl-2-*H*-tetrazolium bromide
DMSO	dimethyl sulfoxide
