# Supplementary material for: 1,10-Secoguaianolides from Artemisia austro-yunnanensis and Their Anti-Inflammatory Effects
Source: Molecules. 2018 Jul 5;23(7):1639. doi: 10.3390/molecules23071639 (PMC6099792; doi:10.3390/molecules23071639)
Supplement: Supplementary file 1 [file molecules-23-01639-s001.pdf]

1,10-secoguaianolides from *Artemisia austro-yunnanensis* and their Anti-inflammatory effects

Lan Liu, Weifeng Dai, Cheng Xiang, Jun Chi, Mi Zhang*

Faculty of Life Science and Technology, Kunming University of Science and Technology, Kunming 650500, China

Supporting Information

Table S1 The Symbols and Definitions of Calculated Molecular Descriptors of Compounds **1-7**

Figures S1−7 1D and 2D NMR spectra in $CDCl_3$ and HRESIMS spectrum in CH_3OH of **1**

Table S1. The Symbols and Definitions of Calculated Molecular Descriptors of Compounds **1-7**

Symbol	Definition
E3p	3rd component accessibility directional WHIM index / weighted by atomic polarizabilities
L2m	2nd component size directional WHIM index / weighted by atomic masses
E_ang	Angle bend potential energy.
ASPAN	The average span R
P3m	3rd component shape directional WHIM index / weighted by atomic masses
P3e	3rd component shape directional WHIM index / weighted by atomic electrotopological states
ASA	Solvent-accessible surface areas
vsurf_D8	Hydrophobic volume at -1.6
vsurf_DW12	vsurf_EWmin1, vsurf_EWmin2 distance
vsurf_ID8	Hydrophobic integy moment at -1.6
PM3_dipole	The dipole moment calculated using the PM3 Hamiltonian [MOPAC]
MoRSEC21	3D-MoRSE - signal 21 / weighted by atomic charge
MoRSEC29	3D-MoRSE - signal 29 / weighted by atomic charge
MoRSEE4	3D-MoRSE - signal 04 / weighted by atomic Sanderson electronegativities
MoRSEE27	3D-MoRSE - signal 27 / weighted by atomic Sanderson electronegativities
MoRSEM16	3D-MoRSE - signal 16 / weighted by atomic masses
MoRSEM18	3D-MoRSE - signal 18 / weighted by atomic masses
MoRSEM10.1	3D-MoRSE - signal 10.1 / weighted by atomic masses
MoRSEM20.1	3D-MoRSE - signal 20.1 / weighted by atomic masses
MoRSEM22.1	3D-MoRSE - signal 22.1 / weighted by atomic masses
MoRSEN26	3D-MoRSE - signal 26 / weighted by atomic number
MoRSEU8	3D-MoRSE - signal 08 / unweighted
MoRSEU22	3D-MoRSE - signal 22 / unweighted
MoRSEV25	3D-MoRSE - signal 25 / weighted by atomic van der Waals volumes
MoRSEV26	3D-MoRSE - signal 26 / weighted by atomic van der Waals volumes
RDFU10	3D-RDF - signal 10 / unweighted
RDFM13	3D-RDF - signal 13 / weighted by atomic masses

RDFC20	3D-RDF - signal 20 / weighted by atomic charge
RDFC4	3D-RDF - signal 4 / weighted by atomic charge

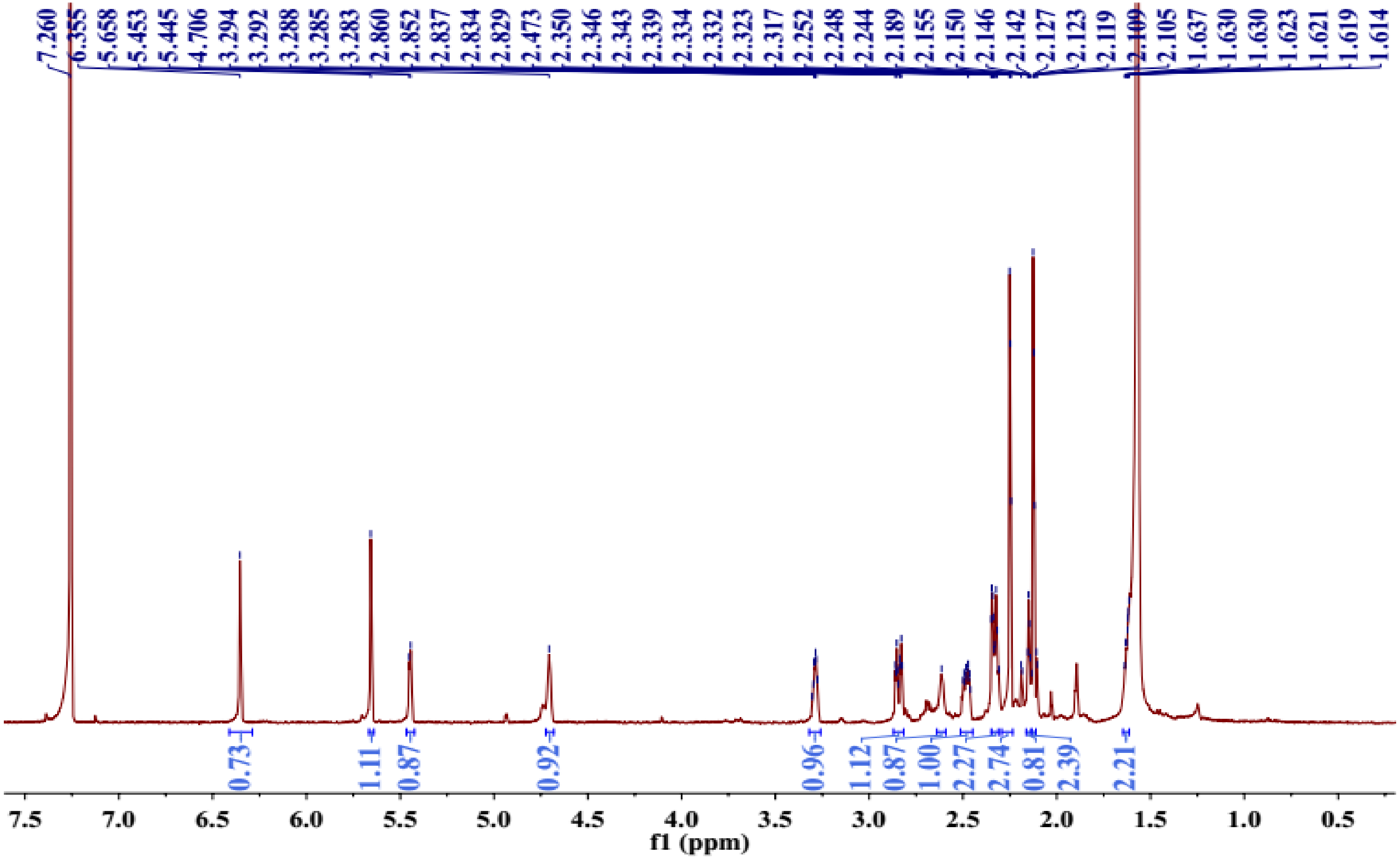

Figure S1 ^{1}H-NMR spectrum of compound **1** in $CDCl_3$

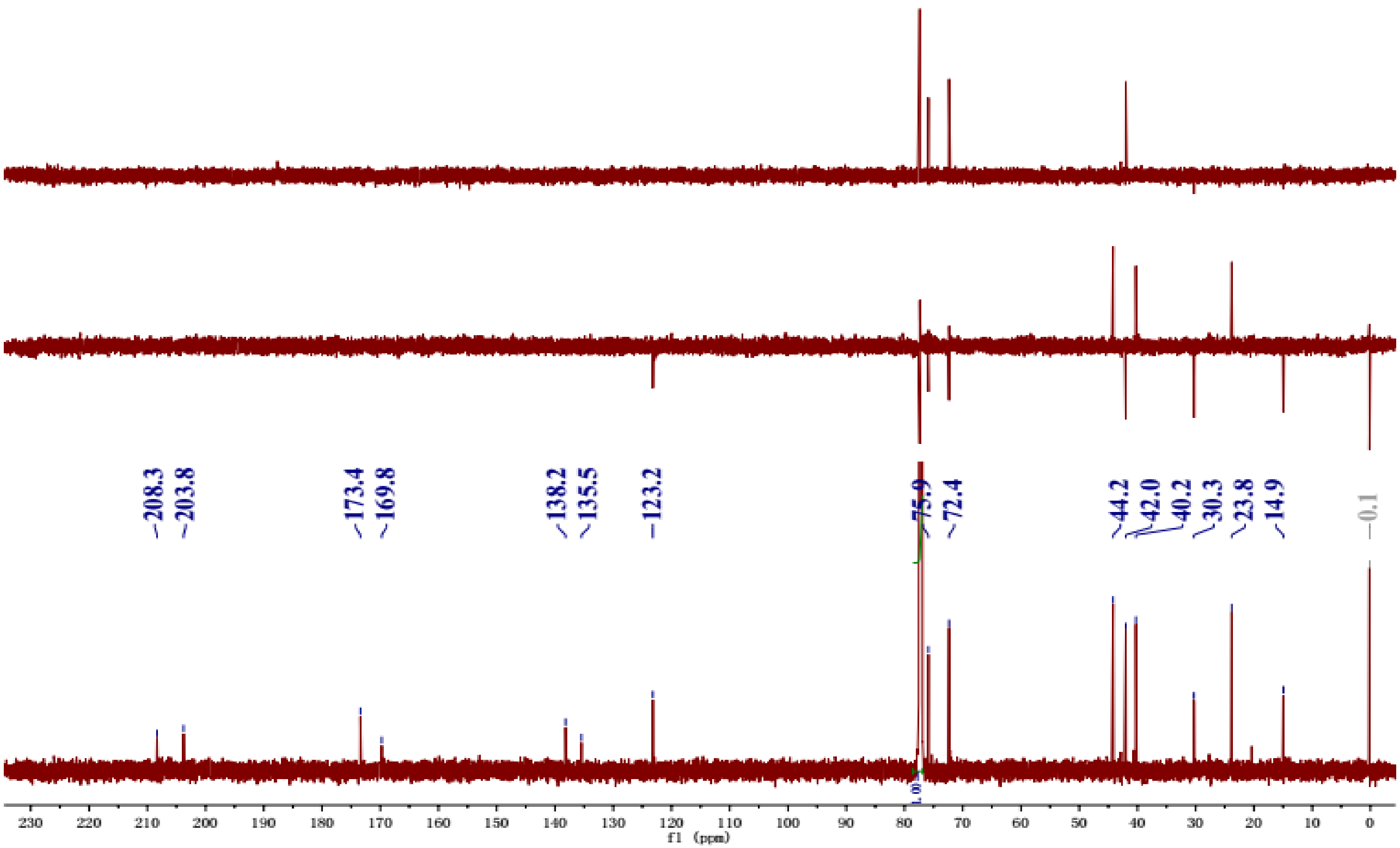

Figure S2 ^{13}C-NMR and DEPT spectra of compound **1** in $CDCl_3$

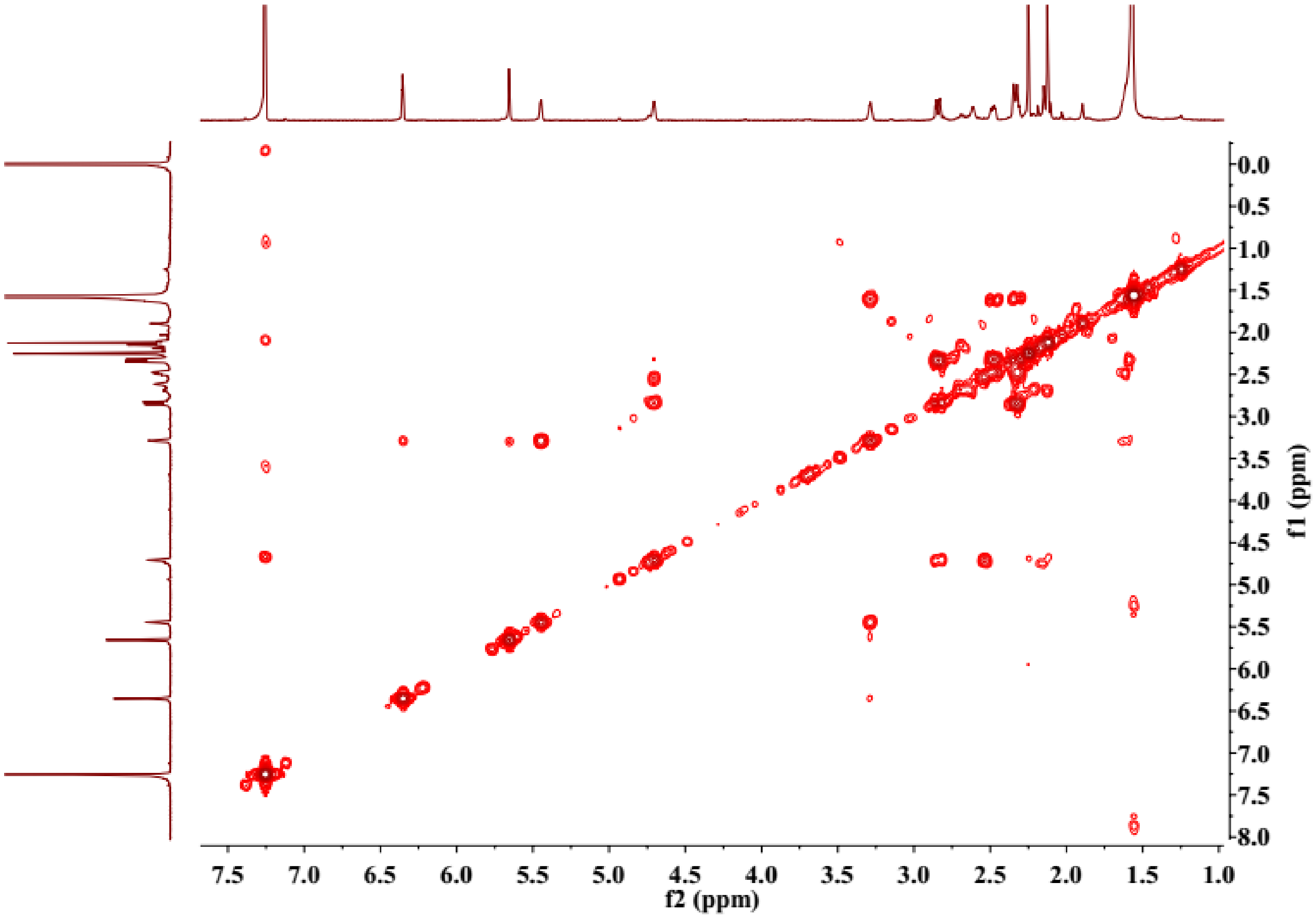

Figure S3 ^{1}H-^{1}H COSY spectrum of compound **1** in $CDCl_3$

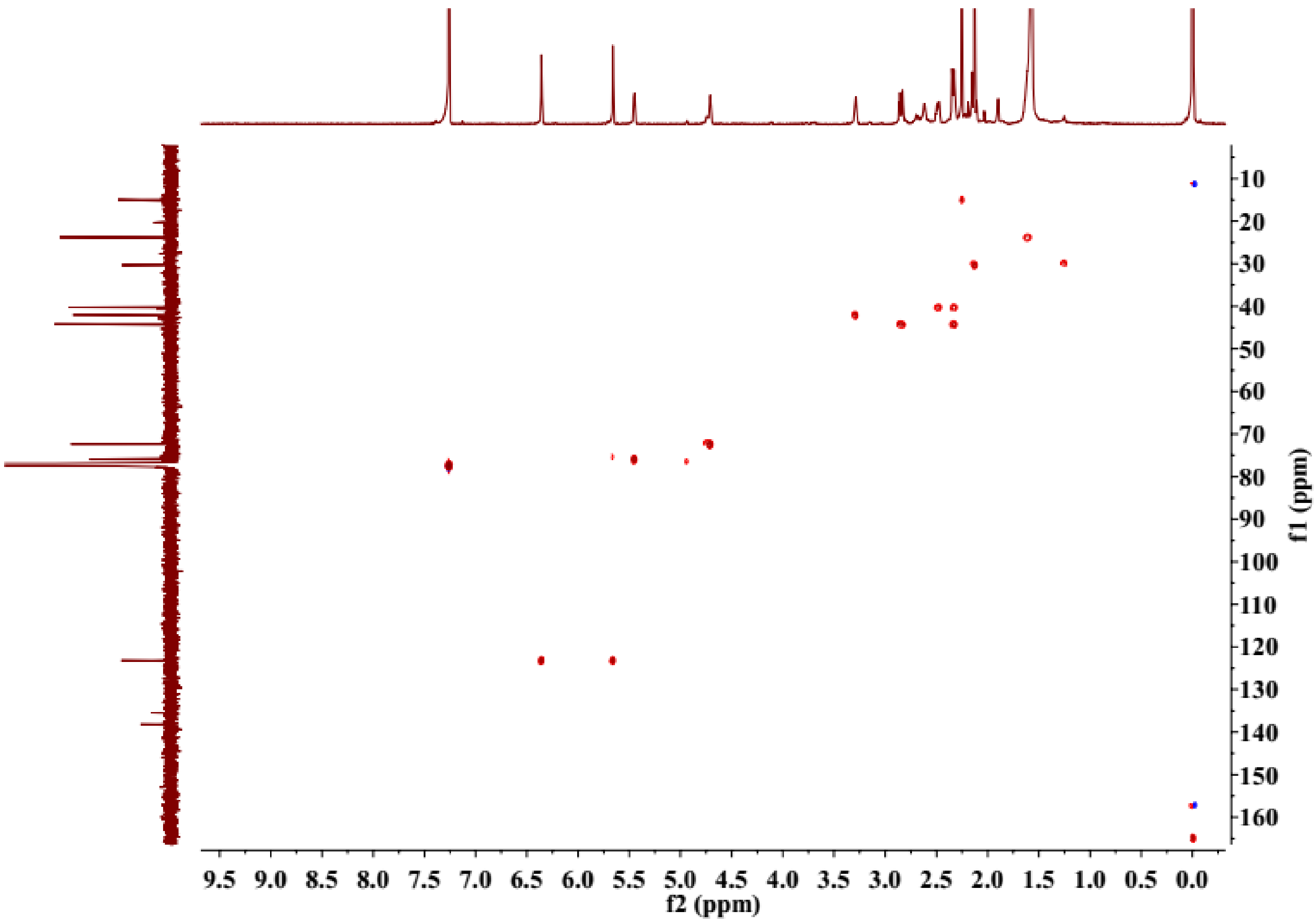

Figure S4 HSQC spectrum of compound **1** in $CDCl_3$

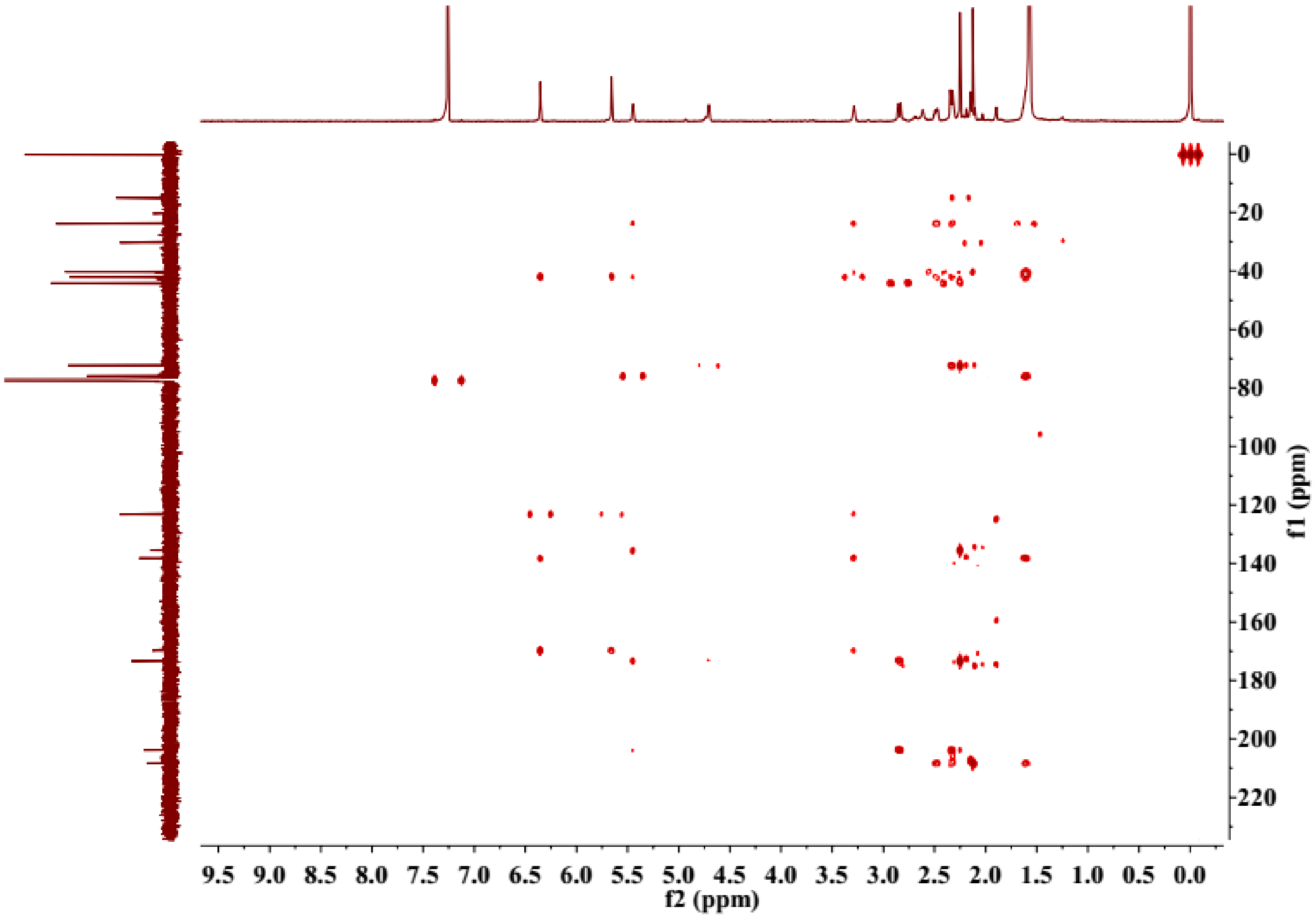

Figure S5 HMBC spectrum of compound **1** in $CDCl_3$

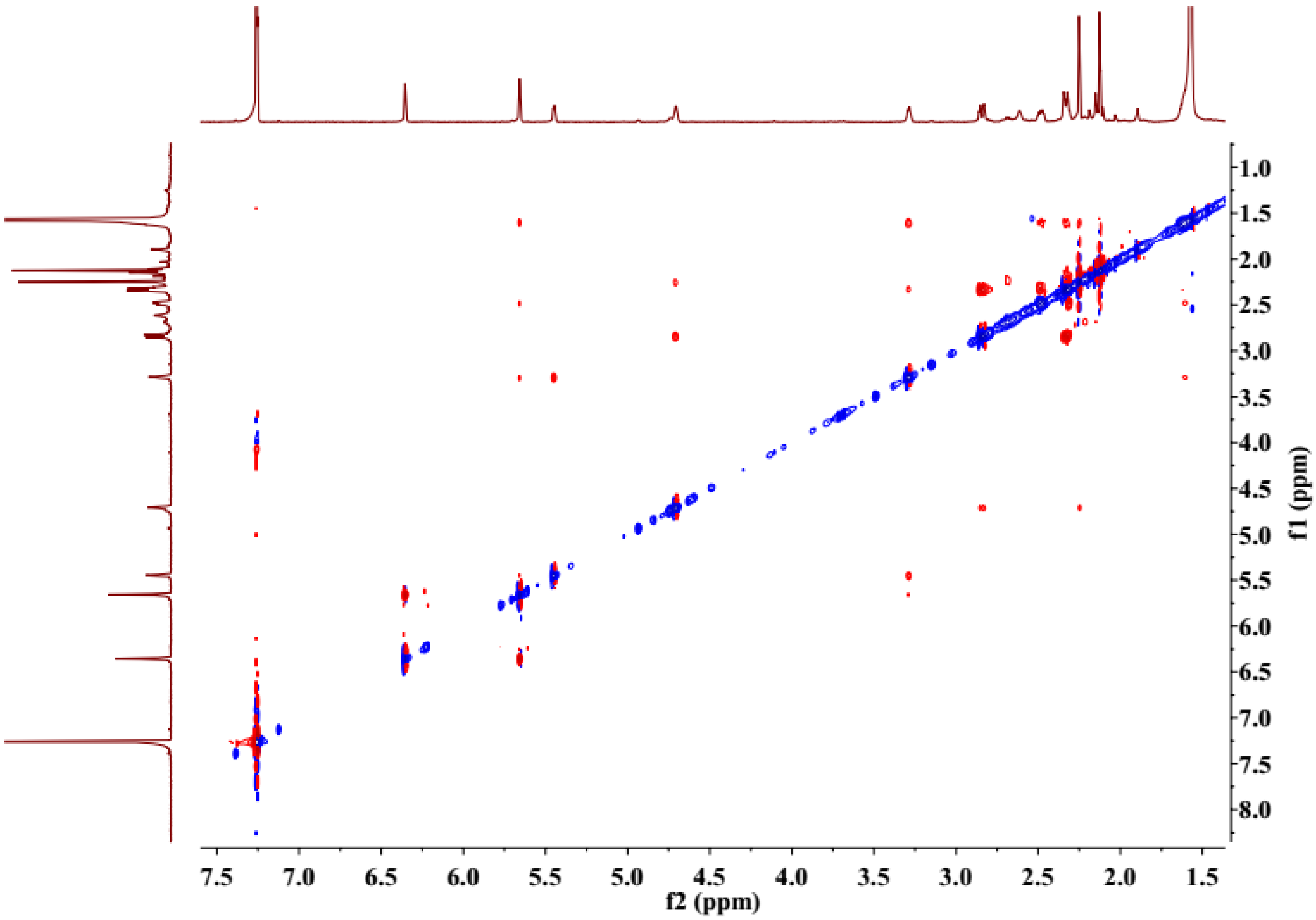

Figure S6 ROESY spectrum of compound **1** in $CDCl_3$

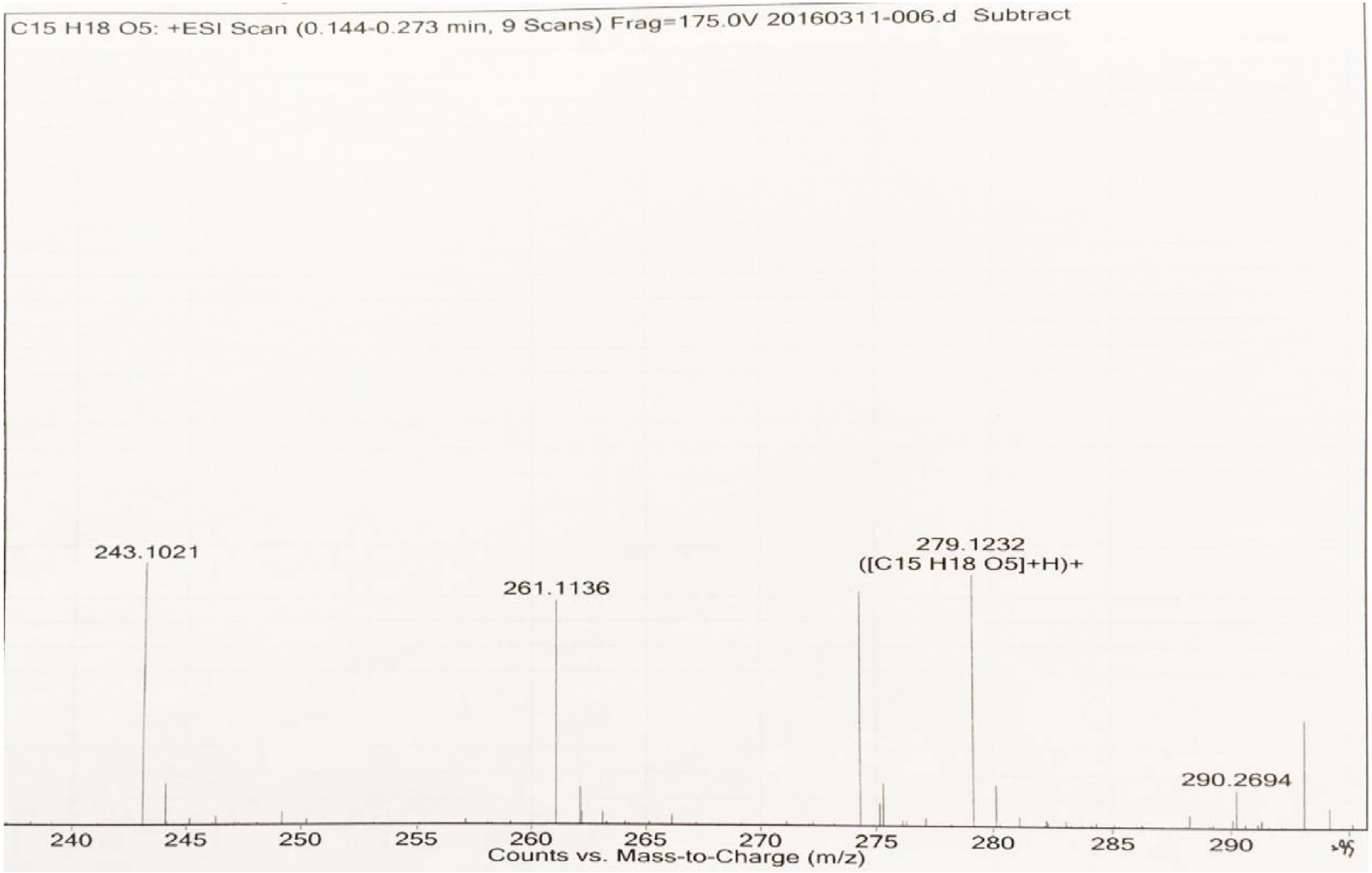

Figure S7 HRSEIMS spectrum of compound **1** in CH_3OH
